# Study protocol on establishment of sentinel sites network for contraceptive and abortion trends, needs and utilization of services in Zika virus affected countries

**DOI:** 10.1186/s12978-017-0282-9

**Published:** 2017-02-02

**Authors:** Moazzam Ali, Kelsey Miller, Rachel Folz, Brooke Ronald Johnson, James Kiarie

**Affiliations:** Department of Reproductive Health and Research, World Health Organization, Avenue Appia 20, Geneva 27, CH-1211 Switzerland

**Keywords:** Contraception demand, Sentinel Sites, Zika virus, Family planning, Safe abortion

## Abstract

**Background:**

ZIKV(Zika Virus) during pregnancy can result in many adverse events such as fetal deaths or newborns with congenital abnormalities including microcephaly and other neural irregularities. Due to these harmful outcomes of pregnancy associated with the Zika virus, we can expect to see a change in the type and scale of demand for family planning and safe abortion services in areas affected by the Zika virus. The monitoring and reporting capacities of the local health clinics in these areas could benefit from the introduction of infrastructural improvements necessary to establish a sentinel site network. Through these sites, the WHO will collect data on the situation from local health professionals to get real time information from the population group and act accordingly to mitigate the consequences of the Zika virus outbreak in a localized and culturally appropriate way. The objectives are to establish a sentinel sites surveillance network for reporting on uptake and utilization of contraception and safe abortion care services; to strengthen monitoring, and data quality assurance in the selected sentinel surveillance sites; and finally to assess the contraception and safe abortion care service utilization trends in the affected sites on a regular basis.

**Methods:**

The proposal includes a set of objectives and actions that enable the creation of a set of criteria for the selection of the sentinel sites, as well the implementation of monitoring and reporting systems that will be used in data collection.

**Discussion:**

The data collected will be used to better understand the changing demand for family planning and safe abortion needs. This will ultimately be used to inform local health workers and policy makers as to how best to track the continued Zika virus outbreak and mitigate the consequences. The learning from establishment of surveillance sentinel sites will help to strengthen health systems at regional and subregional levels that are more adaptable and capable of providing reproductive healthcare services and of responding to future emergencies.

## Plain english summary

The Zika virus’ presence poses a significant threat to women of child-bearing age, and women who are or may become pregnant in the foreseeable future. Some Latin American health officials have recommended to postpone or avoid pregnancy and the WHO has stressed the continued importance of family planning and safe abortion availability. Because these services and products are not always readily available, more research is needed to identify coverage gaps and bolster infrastructure to meet the growing and changing demand for these services. This will be done through the establishment of a sentinel site network. This paper describes the function and historical significance of sentinel sites, and the justification for the establishment of a network in Zika virus affected areas. The proposal includes the establishment of strengthened monitoring and reporting systems, as well as emphasizing education of focal people in local health clinics in the affected areas. Information will be collected via monthly reports, and shared in a dissemination report.

This study will inform policy makers for the purpose of making long-term sustainable changes to strengthen the delivery of contraception and abortion services, including treatment of complications and provision of post abortion contraception care, sustainably and beyond the timeframe of this project.

Establishment of a sentinel site network in the selected Zika virus affected areas will work as a model and will help to prepare the response and mitigate the negative impacts of the current Zika virus response and/or for improved capacity of the health system to respond to community health care needs in sexual and reproductive health for future disease outbreaks.

## Background

The presence of the Zika virus in the Americas is an ongoing concern, and, as infection during pregnancy has been linked to microcephaly, Guillain-Barré syndrome, and other developmental disorders in fetuses [1–3]. It poses a threat to women of child-bearing age, and women who are, or plan to become, pregnant. In February of 2016, the WHO officially declared the Zika virus outbreak to be a public health emergency [4] encouraging many countries in the affected areas to continue their preventative and protective actions for the populations at risk.

Prior to this, some Latin American health officials had already recommended a delay or postponing of pregnancies [5]; officials in some countries, like El Salvador and Columbia, have urged avoiding pregnancy outright [6], while other countries had provided timelines regarding pregnancy avoidance. In Brazil and Ecuador, women have been recommended to indefinitely avoid pregnancy, while other countries have recommended various time limits; women were previously encouraged to postpose for 6–8 months in Columbia, up to a year in Jamaica, and until 2018 in El Salvador [7]. The WHO, while not recommending the avoidance of pregnancy in the context of the Zika virus, recommended the provision of emergency contraceptive services and counselling to all women that are at risk, as well as the continuation of safe sex practices, access to family planning and access to emergency contraception and counselling [8].

While these recommendations vary slightly, they are similar in highlighting the necessity for the availability of family planning products and contraceptive services to avoid or delay pregnancies in the wake of a continued outbreak. However, of the areas that are most affected by the virus, few offer universal access to family planning services [9], and often have limited access to contraception and insufficient information to currently pregnant women about the potential consequences of Zika virus infection, or how to obtain an abortion, when such services is legal [10]. In other cases, pregnancies are unplanned or the result of sexual violence, as sex is not always a choice for women in these areas [11]. The advice to avoid pregnancy is controversial and difficult to follow, especially in contexts where abortion is largely illegal and contraception is difficult to acquire.

Due to the official recommendations and consequences regarding Zika virus and pregnancy, many women will likely want to postpose or avoid pregnancy in these areas, leading to an increase in demand for contraception. Additionally, we can postulate that, without regular access to contraception, women will increasingly seek abortion and post-abortion care. Indeed, the shift and increase in demand is already happening- in November 2015, after PAHO (Pan American Health Organization) released a health advisory declaring the Zika virus a health emergency in Latin America, a study showed that the demand and requests for abortions in Latin American countries increased [12]. In the context of a crisis like the Zika virus outbreak, women will likely continue to seek out abortions and contraception methods, but, without safe and legal access to meet their demands, women will be driven to seek unsafe abortion and continue to have unplanned pregnancies.

### Inequality of access to family planning in the context of humanitarian crises

The Ebola outbreak showed that many health systems are vulnerable and can quickly break-down in the wake of a humanitarian crisis. Health centers in Liberia, Sierra Leone, and Guinea were overwhelmed by the Ebola outbreak, and, as the health system failed, many people could not obtain the basic health care that they needed, and women in need of reproductive and obstetric care suffered [13]. As we saw with Ebola, health centers can become overwhelmed and fail to meet the basic needs of its clients, and women in particular are disadvantaged, especially in the context of sexual and reproductive health. In order to avoid another health system breakdown, countries in Zika virus affected areas should anticipate an increased demand for family planning and contraception, and bolster their preparedness plans accordingly.

Additionally, inequality of access to health care plays a role in the Zika virus outbreak. Those that will be most likely negatively affected by the Zika virus are the poorer populations that are generally subject to health care inequalities and poorer health. These at-risk populations tend to have a disproportionate lack of access to quality health care, and are at a greater risk of infection and poor health due to poor sanitation, lack of clean water, and overcrowding. Additionally, public health programs in these areas tend to be more readily available and accessible to people of higher socioeconomic status before those of lower status, thus contributing to the cycle of poor health and inequality [14].

### Sentinel surveillance sites - experiences from the field

A ‘Sentinel Site’ is a healthcare facility with a specified geographical catchment area that is the focus of ‘place-based’ Sentinel Sites Evaluation activities. Sentinel surveillance activities are often established to ensure high-quality data for a particular disease or intervention in limited resource settings and the resulting analysis is used to inform programs and policies affecting a larger geographic area. Many countries have developed surveillance activities for communicable diseases in order to monitor disease burden, detect outbreaks of epidemic-prone diseases and monitor progress towards national or international control and eradication targets. Similar systems can be used in situations where services may be overwhelmed by demand. Whereas most passive surveillance systems receive data from as many health workers or health facilities as possible, a sentinel surveillance system deliberately involves only a limited network of carefully selected reporting sites. The feasibility of sentinel surveillance has led WHO to recommend that countries adopt the approach for monitoring interventions and programs [15].

Sentinel sites, and their consistent monitoring and reporting play an active role in the Polio Global Eradication Initiative as seen in their technical reports; these sites are encouraged to have proper information dissemination, effective education of staff, contextually appropriate placement in a country, and adequate reporting techniques [16].

Sentinel sites, while associated closely with polio, have also been employed to combat other public health challenges. In Vietnam, during 1998–2003, sentinel sites were established at 6 hospitals across 4 cities with the support of the WHO to track Rotavirus in young children; the sentinel sites proved to be a success in that, with constant surveillance and dedicated supervision they were able to provide detailed and reliable data in a timely matter, all the while being cost effective [17].

Sentinel sites can also be used in a non-virus context, and used instead to track emerging public health trends. For example, during 2008–2012 India had relative success in setting up and monitoring anti-malarial drug resistance with a series of 25 sentinel sites. The sites were found to be an effective monitoring method to cover wide geographical areas, track trends, and communicate effectively [18].

Recently, a report suggests that the use of already existing surveillance systems to track acute flaccid paralysis (AFP) is key to tracking potential Zika virus cases because of its relation to Guillain-Barre syndrome; Guillain-Barre, while classified as a non-polio AFP case, is the most common non-polio cause of AFP, and is also linked to some cases of Zika virus infection [19].

This project intends to understand the changing needs and demands for contraception and safe abortion, including treatment of post-abortion complications and provision of post abortion contraception in Zika virus affected countries while also identifying weaknesses in the healthcare system and the capacities of healthcare workers to respond to the crisis under way. This project will also include a training element to help health care workers develop better reporting and monitoring skills, as well as data-related skills to aid in the rapid collection and transmission of quality data to the Ministry of Health in their area, creating a network of workers able to properly collect and process data. Further to this aim, WHO will provide a short orientation course on the latest guidelines in contraception to the healthcare providers on the frontline to identify, prescribe and deliver methods of choice through training in both research and service delivery. Finally, data derived from this project will inform policy makers for the purpose of making long-term sustainable change to strengthen the delivery of contraception and safe abortion services, including treatment of complications from unsafe abortion and provision of post abortion contraception, sustainably and beyond the timeframe of this project.

### Study aims and objectives

The main aim of the project is to assess and strengthen health care system response to changing needs and demands for contraception and safe abortion in emergency situations, specifically Zika-virus affected countries. Additionally, it will help to identify any weaknesses in the healthcare system or the capacities of healthcare workers to respond to the crisis underway.

The objectives of this project are threefold.

The first objective is to identify and establish a sentinel site surveillance network from pre-existing hospital sites for reporting on uptake and utilization of contraception and safe abortion services in partnership with the ministry of health. The second objective is to strengthen monitoring and reporting in health care for the selected sentinel surveillance sites, specifically for contraception and safe abortion services. The third objective is to develop and strengthen the data-related activities in order to assess the contraception and abortion service trends in the affected sites on regular basis.

## Methods/Design

The sentinel sites will be established in ZIKV affected areas in collaboration with the Ministries of Health in Brazil, Honduras, and Columbia. These areas will be identified in collaboration with local authorities and with the cooperation of the state’s Ministry of Health.

The sentinel sites will be established following a three stage process. The steps have been developed by the WHO team in collaboration with partners from the Ministry of Health, and are as follows:

- Step 1. Mapping of facilities in ZIKV-affected areas
  All facilities in selected ZIKV-affected areas with the potential to provide services in contraception and safe abortion in selected countries i.e., Brazil, Honduras and Columbia will be identified and mapped in collaboration with Ministry of Health.
- Step 2. Assessment of health facilities
  Following step 1, we will conduct a facility assessment in ZIKV-affected areas on the provision of contraception and safe abortion in the selected countries using validated WHO tools. This activity will be reported in detail separately in a forthcoming article.
  Through the facility assessment, we will gather information on the types of contraception and abortion services available in local health centers, and assess the local health authorities’ readiness and qualifications in addressing concerns and providing accurate information to clients/patients of all socioeconomic statuses. The facilities will be assessed using validated tools developed by WHO.
- Step 3. Identification of sentinel surveillance sites
  The suitable sites, based on the result of above-mentioned assessment, will be identified in collaboration with MoH, and be designated as sentinel sites. Infrastructure support will be provided on the study tool, data collection and data entry, monitoring, quality assurance and data management. There will be comprehensive training for focal persons on data collection and reporting systems at selected sentinel sites.

Once established, the sentinel sites will monitor and measure contraception and abortion service uptake and trends in populations in the affected countries. The sentinel sites will collect information on contraceptive method mix and provision, abortion, and the demographic and socioeconomic status of users. Information will also be collected on method availability and staffing at the facilities providing contraception and abortion services.

The information from the sentinel sites will inform improvements on the implementation of reproductive health programs that include contraception and abortion, identify how the programs could address barriers to services, trends in demand of services and facilitate effective implementation of programs at the local level to meet the needs of the target population.

### Deliverables

Key deliverables for the project will include a network of sentinel surveillance sites in Zika virus affected areas in selected countries, as well as an implementation framework and a set of key indicators on changing demand, capacity, needs, and trends in family planning and safe abortion. Key inputs will also include the development of monitoring and data collection tools.

Key outputs will be largely based on the collection of data on multiple factors, including the demand of contraceptive and abortion services. The availability of the contraception available and the range of services will also be assessed, as well as the barriers to accessing contraception and safe abortion services. Data will also be collected on the demographic and socioeconomic status of the clients/patients using the services. There will also be information collected regarding the development of selection criteria and characteristics of sentinel sites for contraceptive and abortion services, as well as information on the personnel providing the services. The collected data will help to identify the trends in contraception and abortion demands and clients’ use of services, and identify areas of weakness or strength in the existing health centers.

### Quality assurance

The principal investigator and project team will be trained on the study purpose, design, quality assurance, reporting mechanisms and data collection tools. Data collectors will also be trained to use the study questionnaire/tool for data collection from the facilities and on entering the survey data in an online tool OpenClinica. Surveyors will be trained on data collection, transmission, verification, storage and primary analysis to assess errors.

All data will be entered into OpenClinica software tool by trained data operators. Measures will be taken to ensure the quality of collected data. All the forms will be checked on a daily basis for completeness, logical errors, and unclear or irrelevant responses. The principal investigator, co-investigator and project team will make monitoring visits to ensure the quality of data and adherence to the study protocol. If needed, refresher training will be arranged to enhance understanding of data collection tools.

### Scientific and ethical review

Scientific review and approval of the proposal has already been obtained from Research Projects Review Panel, the external review body of the Department of Reproductive Health, and Research (WHO/RHR) including the UNDP/UNFPA/WHO/World Bank Special Programme of Research, Development, and Research Training in Human Reproduction (HRP). Ethical approval for facility assessment that will lead to identification of sentinel sites has also been approved by WHO Research Ethics Review Committee.

### Timeline of project

We anticipate that the study will take 24 months. The initial stage included the development and finalization of the concept note and the research proposal, technical and ethics clearance as well as the initial identification of sentinel sites. These activities will take place during Quarter I, in Brazil, with collaboration between WHO and the regional and county offices of the Ministry of Health.

The second phase of the project will occur in Quarter II, and will consist of the development of the tools, data-entry software, and the establishment of reporting protocols for the projects, including monitoring framework, indicators, standard operating procedures and reporting forms, data collection forms and reporting mechanisms. The second component in Quarter II is the training of the sentinel site focal persons. This training will include data collection and recordkeeping, information reporting techniques (including timing and modality), and the establishment of the data quality control mechanism. While these activities are the main focus of this training phase, there are secondary skills that will be strengthened through the training. These secondary skills include the strengthening of contraceptive counselling using WHO guidelines, and service provision in LARC and emergency contraception. These activities will be performed by WHO and the collaborating partners at the Ministries of Health.

Beginning in Quarter III, the focus of the project will shift to data collection and data entry, using the introduced methods and guidelines. A major component of this phase will be the routine monthly clinic service record report, which will be eventually aggregated to quarterly reporting data. This will be stated in the initial sites in Brazil, and eventually be started in other locations. These activities will be carried out primarily by the sentinel site focal staff, with support from the WHO regional offices and the respective ministries of health.

After this shift to data collection in Quarter III, the project will move to a quarterly reporting phase which included validation of the quality of the data collected and follow up procedures of the sentinel sites based on reported data. These activities will take place quarterly and include quarterly status reports, reporting teleconferences, and feedback regarding implementation and any associated issues. These activities will be carried out by WHO, sentinel site focal persons, and the Ministry of Health. Additionally, there will be monthly analysis and report writing, and will include monthly reports on data collection, and quarterly report analysis at the Ministry of Health.

The dissemination process will occur at the end of the Quarter III and throughout Quarter IV. The aim of this dissemination is to assess the project on the ground, and adjust the reporting mechanism based on continuing lessons learned. This will be performed by the WHO regional offices and the Ministry of Health.

Finally, in the second year, identification and implementation of the sentinel sites will begin in El Salvador and Columbia, with a similar pattern of activities.

## Discussion

This paper describes the project proposal to create a network of sentinel sites to aid in the capacity building of affected areas to meet family planning and safe abortion needs in the context of the Zika virus outbreak. Our objective is to firstly anticipate the potential shift and increase in demand for family planning/contraception needs and safe abortion, and then to establish an integrated and functional network of sentinel sites in the affected areas for obtaining real time information from the field.

In long term, the data derived from this project will provide evidence to strengthen contraception and safe abortion policies that will lead to sustainable change that will endure beyond the timeframe of this project. Establishment of a sentinel site network in the Zika virus affected areas will work as a model and will generate substantial impact for the current Zika virus response and/or for improved capacity of the health system to respond to community health care needs in sexual and reproductive health for future disease outbreaks.

## Abbreviations

LARC: Long-acting reversible contraception
SSE: Sentinel sites evaluation
TORs: Terms of reference
WHO: World Health Organization
