# Correction to: Study protocol on establishment of sentinel sites network for contraceptive and abortion trends, needs and utilization of services in Zika virus affected countries

**DOI:** 10.1186/s12978-017-0432-0

**Published:** 2017-12-19

**Authors:** Moazzam Ali, Kelsey Miller, Rachel Folz, Brooke Ronald Johnson, James Kiarie

**Affiliations:** Department of Reproductive Health and Research, World Health Organization, Avenue Appia 20, CH-1211 Geneva 27, Switzerland

## Correction

The original version of this article [1] unfortunately contained a mistake. All occurrences in the main text referring to the research carried out in the following countries: Brazil, Honduras and Columbia should instead be replaced with “Brazil, Honduras and Panama”. The original version of this article has been updated to reflect this change.
